# Provider Perceptions of Over-the-Counter Progestin-Only Birth Control Pills in Urban, Suburban, and Rural Populations of Pennsylvania

**DOI:** 10.7759/cureus.94175

**Published:** 2025-10-09

**Authors:** Schyler Said, Sarah Waszyn, Katelyn D Le, Xuezhi Jiang

**Affiliations:** 1 Obstetrics and Gynecology, Drexel University College of Medicine, Philadelphia, USA; 2 Obstetrics and Gynecology, Reading Hospital/Tower Health, Reading, USA

**Keywords:** birth control options, healthcare provider attitudes, opill, over-the-counter birth control, progestin-only pill

## Abstract

Background

The Opill is an over-the-counter, progestin-only oral contraceptive and the first of its kind to free women from the need to obtain a prescription for birth control. Opill is approved in Pennsylvania, a remarkably diverse state containing urban, rural, and suburban regions spanning the state. As women gain easier access to birth control that does not require a medical provider's prescription, the Opill may help overcome healthcare gaps when seeking birth control methods. It is crucial to understand provider opinion on progestin-only pills (POPs), which influence patient education and use of the progestin-only pills to prevent unintended pregnancies. In this study, we aim to survey providers who prescribe birth control in Pennsylvania's rural, urban, and suburban regions to gain insight into their opinions of the Opill.

Methodology

The surveys were emailed to healthcare providers in OBGYN, family medicine, internal medicine, and pediatrics using Drexel University College of Medicine’s affiliated clinical sites throughout Pennsylvania. The provider’s demographics, knowledge, and attitudes toward Opill were collected.

Results

The survey was sent to 764 providers, and 60 (8%) completed it. Fifty-one (85%) participants did not receive any information from their institution regarding over-the-counter birth control pills, and 36 (61%) received no formal training on patient counseling for over-the-counter birth control. Unawareness of the availability of Opill among rural, suburban, and urban providers was 3 (75.0%), 11 (34.5%), and 6 (27.3%), respectively.

Conclusions

There is a notable lack of awareness and clinical training of the Opill within Pennsylvania's urban, suburban, and rural providers. Because of the lack of training, providers may be less likely to support and recommend Opill to patients as opposed to other oral contraceptives. As Opill continues to gain traction in the market, further studies are needed to determine whether provider training, perceptions, and recommendations regarding Opill will change over time.

## Introduction

Opill represents a significant advancement in contraceptive options available. As an over-the-counter, progestin-only oral contraceptive, Opill is the first of its kind to free women from the need to obtain a prescription for hormonal birth control [1]. Opill has been approved in the state of Pennsylvania for release in 2024. Pennsylvania is diverse in urban and rural areas, and both extremes also have difficulty with access to care [2]. Opill represents a promising option for these populations; however, the increased access it hopes to provide will be affected by physicians all the same [3,4].

The introduction of Opill in Pennsylvania is poised to be a significant milestone in the realm of reproductive health [2]. By offering a progestin-only pill over the counter, Opill seeks to bridge gaps in contraceptive access exacerbated by geographical, socioeconomic, and political barriers. This could be particularly transformative in the more isolated rural areas where healthcare facilities are sparse and in urban areas where the demand for reproductive health services can lead to long waits and delayed care [5-7].

The anticipated impact of Opill also extends to the domain of healthcare provider-patient dynamics. Traditionally, the prescription model has positioned physicians as gatekeepers to birth control, creating an inherent power dynamic that Opill could disrupt. The availability of Opill could potentially alter how physicians approach contraceptive counseling, shifting the emphasis towards education about self-managed options [6-8]. However, physicians' attitudes toward and knowledge of Opill will be a crucial determinant of its successful integration into existing contraceptive care models [4,5]. Their support or resistance could significantly influence patient uptake and, therefore, the overall effectiveness of Opill in enhancing reproductive autonomy.

This study aims to survey providers across Pennsylvania in varying practice settings (rural, suburban, and urban) on their comfort level with and knowledge of progestin-only birth control pills and specifically over-the-counter Opill.

## Materials and methods

Study procedures included one online, voluntary, and anonymous survey through Qualtrics delivered to providers (MD, DO, NP, PA) in specialties that typically counsel on birth control - family medicine, obstetrics and gynecology, pediatrics, and internal medicine. The full survey is included in Appendices A-G.

The Qualtrics survey presented with the following categories: screening questions, provider demographics, provider training and education, perceptions of patient volume/demographics, provider perception of birth control barriers, comfort level on prescribing birth control/progestin-only pills, prescribing and counseling Opill, concerns with Opill, and POP misconceptions (in the form of a true or false quiz). Questions assessing provider attitudes on Qualtrics were asked in a 4- and 5-point Likert scale.

Providers were recruited to fill out the survey by an email invitation delivered to providers in family medicine, obstetrics and gynecology, pediatrics, and internal medicine in healthcare institutions associated with Drexel University College of Medicine (DUCOM). This study was approved by the Institutional Review Board of DUCOM (Approval No. 2401010345). Informed consent was obtained from all participants. Clinical directors of each DUCOM-associated site were asked to forward the survey to their hospital list-serves and provide the number of providers in their department. For clinical directors who failed to respond, follow-up emails were sent out each week requesting that to forward the email invitation to their providers. Reminders were sent out every four weeks during the duration of the study. The recruitment period went on for three months, during which all subjects may voluntarily self-enroll by electing to complete the survey.

Quantitative data were analyzed through parametric and non-parametric methods in SPSS Version 29.0.2.0 (IBM Corp., Armonk, NY). Categorical data were compared using the Chi-square test, and continuous data with normal distribution were analyzed by Student’s t-test. Non-parametric Mann-Whitney U tests were applied to ordinal data or non-normally distributed continuous data.

The study focused on provider perceptions to represent patients in this project. As many patients receive health information through their providers, provider knowledge and attitudes are the first steps in patient understanding of Opill as a reliable form of contraceptive.

## Results

Demographics

Characteristics of Respondents

The survey was sent to 764 providers across the state of Pennsylvania and completed by 60 (8%) providers. The sample represented various practice settings, provider types, specialties, and practice types. The demographics of participants are detailed in Table 1.

**Table 1:** Demographics of respondents.

Characteristic	Category	Count	Percentage
Practice setting	Rural	4	6.67%
	Urban	24	40%
	Suburban	32	53.33%
Provider type	Physician (MD/DO)	50	83.33%
	Nurse Practitioner	6	10%
	Physician Assistant	0	0%
	Other	4	6.67%
Specialty	Obstetrics and Gynecology (Ob/Gyn)	16	26.67%
	Family Medicine	24	40%
	Internal Medicine	8	13.33%
	Pediatrics	11	18.33%
	Other (Midwifery)	1	1.67%
Practice type	Private	6	10%
	Hospital	39	65%
	Other	25	25%
Institution type	Academic	37	61.67%
	Community	20	33.33%
	Private Practice	3	5%

Comparison of Providers by Practice Setting

Rural providers:Of rural providers, only 1 (25%) reported receiving formal education on over-the-counter contraceptives. Despite this, 4 (100%) respondents did indicate that they knew that Opill was available over-the-counter. Half of rural providers reported that most of their population has no medical insurance.

Urban providers: Twelve (52.2%) respondents had received formal training on over-the-counter birth control pills. Most of these providers (17, 70.8%) reported that the majority of their patient population was Medicaid recipients. A significant proportion of these providers (18, 75%) indicated that most or nearly all of their patients have limited access to healthcare. Ten (40%) urban providers were unaware that Opill is available over-the-counter.

Suburban providers:Suburban providers reported the highest rates of lacking formal training, with 21 (65.6%) indicating they had not received any formal education on over-the-counter birth control pills. Despite this, 26 (82.7%) suburban providers expressed comfort in prescribing progestin-only pills. Fourteen (45.1%) reported that most of their patients were underserved or had limited access to healthcare.

A chi-square test of independence examined the association between practice setting and awareness that Opill is available over the counter. The association was not significant, χ²(2, *N* = 60) = 1.21, *P* = 0.547, Cramer’s *V* = 0.14, indicating a small effect size. Because 33% of cells had expected counts less than 5, this result should be interpreted with caution. These findings suggest that awareness of Opill did not significantly vary across practice settings.

A second chi-square test assessed differences in formal training across practice settings. Results were not significant, χ²(2, *N* = 60) = 2.92, *P* = 0.232, Cramer’s *V* = 0.22, suggesting only a small-to-moderate association. This indicates that levels of formal training were relatively consistent across practice settings.

Key findings

Training and Awareness

Of the providers surveyed, 37 (61%) reported receiving no formal education or training on over-the-counter birth control pills, and 51 (85%) had not received information about over-the-counter birth control pills from their institution or practice. Eighteen (30.2%) providers were unaware that Opill is currently available over-the-counter in Pennsylvania.

Provider Comfort and Recommendation

Fifty-one (85.2%) providers surveyed agreed to being comfortable counseling on birth control with patients; however, only 47 (77.8%) felt comfortable prescribing progestin-only pills for patients without contraindications. Thirty-two (54%) of respondents indicated that their patients are uninterested in switching to an over-the-counter progestin-only pill. Twenty-four (40.8%) of providers expressed being unsure about recommending Opill to patients, and 15 (25%) do not recommend it at all.

Patient Demographics and Challenges

Fifty-three (87.7%) of respondents reported that at least some of their patients experience unintended pregnancies. Fifty-two (86%) providers indicated that they treat at least some patients without any medical insurance, and 34 (56.1%) providers reported that most or nearly all of their patient population is Medicaid recipients, and 54 (89.5%) providers indicated that some or most of their patient population is underserved and has limited access to healthcare.

True/false knowledge assessment

Effectiveness

Forty-two respondents (70%) correctly identified that progestin-only birth control was as effective as combination pills when taken correctly.

Weight Gain

Fifty-five (92%) correctly disagreed with the statement that progestin-only pills are associated with a much higher incidence of weight gain than combination pills [10].

Adherence Challenges

Thirty-eight (63.3%) respondents correctly recognized that adherence to progestin-only pills is significantly lower due to their limited efficacy outside a three-hour window [11]. More than one-third of respondents (n = 22) may not have fully understood this.

Contraindications

All respondents (60, 100%) correctly disagreed with the statement that progestin-only pills have a higher occurrence of contraindications compared to combination oral contraceptives [12].

Mechanism of Action

Fifty-six (94%) of respondents correctly understood that part of Opill's mechanism for preventing unintended pregnancies is by thickening the cervical mucus. This high level of knowledge is encouraging, but the remaining 4 (6%) represents a small but important gap [13].

Nonsignificant Differences in Other Group Comparisons

Chi-square analyses were conducted to examine associations between provider type, practice setting, and specialty versus awareness, knowledge gaps, and provider comfort questions. None of the associations was statistically significant. Specifically, analyses comparing practice setting with awareness and related questions yielded χ²(2, *N* = 60) values ranging from 1.21 to 2.92, with Cramer’s *V* values between 0.14 and 0.22, indicating small effect sizes. Comparisons across provider types produced χ²(3, *N* = 60) values of 0.85-2.40, with Cramer’s *V* values from 0.12 to 0.20, while specialty comparisons showed χ²(4, *N* = 60) values between 1.05 and 3.12 and Cramer’s *V* values of 0.13 to 0.23. Across all analyses, effect sizes were small, suggesting negligible associations. These findings indicate that awareness, knowledge, and comfort levels did not meaningfully differ across provider groups, practice settings, or specialties.

## Discussion

This study aimed to assess healthcare provider awareness, knowledge, and comfort regarding over-the-counter contraceptives. The goal was to assess differences across practice settings. Unfortunately, the low number of respondents from rural settings limited the ability to perform robust statistical analysis between these groups. It should be noted that the low number of respondents could be due to some of the providers working for multiple institutions contacted and may have been counted multiple times in the total 764 emailed providers. Despite this, the findings of the study provide valuable insights into the attitudes of providers in the state of Pennsylvania, which may impact the effective adoption of Opill among patients.

Provider knowledge gaps and training needs

A notable 18 (30.2%) providers were unaware that Opill is currently available over-the-counter. This lack of awareness is concerning, given the importance of provider knowledge in guiding patient choices and ensuring safe and effective use of contraceptives. This finding is consistent with previous research indicating that healthcare providers often lack up-to-date knowledge about newly available contraceptive options [14]. As 37 (61%) respondents reported no formal education on over-the-counter birth control pills and 51 (85%) provider respondents had not received any information regarding Opill from their institution, healthcare professionals may need formal training to recommend Opill as a contraceptive option. Provider training is crucial, as studies have shown that provider knowledge and attitudes significantly influence contraceptive use and patient outcomes [15].

The true/false knowledge assessment revealed that while many providers answered most questions correctly, there are some key gaps in knowledge. For example, 18 (30%) respondents were unaware that progestin-only pills can be as effective as combination oral contraceptives when taken correctly. This aligns with previous studies showing that providers often do not have proper knowledge of contraceptive effectiveness [16].

Comfort levels and provider concerns

Most providers (51, 85.2%) reported feeling comfortable counseling on birth control; however, only 47 (77.8%) felt comfortable prescribing progestin-only pills. This discrepancy suggests that providers are hesitant about progestin-only pills. This hesitancy may be due to adherence and efficacy issues, especially in relation to the strict timing requirements of progestin-only pills, which necessitate patients to take the pill at the same time every day for efficacy [11].

As 32 (54%) respondents indicated that their patients are not interested in switching to over-the-counter birth control, and 37 (61%) providers reported that none of their patients have expressed interest or curiosity in Opill, it seems as though the public may just not be interested in Opill. However, the lack of provider knowledge likely indicates a general lack of knowledge surrounding Opill in the community, which may be leading to the low interest among patients. Further research is needed to study how patients can be better informed about Opill as an option. Even more, some of the provider concerns, such as adherence and efficacy issues, may be alleviated by better patient education.

While 15 respondents (25%) did not recommend Opill and 24 respondents (40.8%) were unsure about recommending it, further education is needed to reduce provider apprehension regarding Opill. An increase in educational efforts at the institutional level may allow respondents to be more open to Opill as a contraceptive for their patients. With evidence-based information about over-the-counter progestin-only pills, physicians may grow in support for Opill as an effective use of contraception for patients [17]. This evidence-based information can be provided to healthcare professionals in their medical training or academic institutions to uniformly distribute credible information regarding Opill.

Patient demographics and barriers

Although most provider respondents treat patients with limited access to healthcare, respondents still show a majority of uncertainty in recommending an over-the-counter method of birth control. Fifty-two (86%) respondents treated patients with no medical insurance, 34 (56%) reported that most of their population was on Medicaid, and 54 (89.5%) reported that some or most of their patient population was underserved. Marginalized populations, such as women of color and those under the poverty level, are patients who show the highest rates of unintended pregnancy [17]. Opill has beneficial aims specifically for patients who experience barriers to healthcare [18]. Additionally, according to previous research, patients with a history of barriers to birth control access exhibited an interest in over-the-counter pill use due to the convenience and ease of access of Opill [19]. However, provider respondents in the survey still exhibited uncertainty in recommending Opill to their patients, despite the large majority of patients who experience barriers in healthcare. Thus, there is a discrepancy between provider perception of patient access to care and provider perception of Opill as a reliable option for patients who experience barriers to care.

Study limitations and future directions

This study has several limitations, including the small sample size and the low number of rural respondents. These problems restrict the generalizability of our findings. There may also be response bias, as the data are self-reported, and providers who are more engaged in reproductive care may have been more likely to respond. If this is true, however, it may make our results even more meaningful, in that those who did not respond could be even less educated on Opill. Future studies should aim for a larger and more representative sample size and try to specifically increase representation from rural settings. There is also a need for further research to explore patient perspectives on the over-the-counter contraceptive Opill.

## Conclusions

Despite this study's limitations, the responses reveal significant gaps in provider knowledge and comfort with Opill. These findings emphasize a need for educational efforts and improvements in training, information dissemination, and institutional systematic efforts to update providers on current contraceptive options. Future research should include a larger sample and explore strategies to overcome barriers to the adoption of Opill, particularly in populations of underserved patients.

## Appendices

Appendix A: Progestin-only pill provider survey

Screening Question 

How often do you prescribe or counsel on birth control?

o Very often (multiple times per week) (1)  

o Often (about once per week) (2)  

o Sometimes (once per month) (3)  

o Rarely (few times per year) (4)  

o Never (5)  

Do you have any financial conflicts associated with Opill?

o No (1)  

o Yes (2)   __________________________________________________

Provider Demographics

Q1 How would you describe your practice setting?

o Rural (1)  

o Urban (2)  

o Suburban (3)  

o None of the above 

Q2 What type of provider are you?

o Physician (MD, DO) (1) 

o Nurse Practitioner (2)  

o Physician Assistant (3)  

o Other (4)  

Q3 What is your specialty?

o Obstetrics and Gynecology (Ob/Gyn) (1)  

o Family Medicine (2)  

o Internal Medicine (or subspecialty) (3)  

o Pediatrics (4)  

o Other (5) __________________________________________________

Q4a Which of the following describes your practice type:

o Private (1)  

o Hospital (2)  

o Other (3)  __________________________________________________

Q4b Which of the following describes the institution type your practice is associated with?

o Academic institution (1)  

o Community institution (2)  

o Private practice (3)  (3)

o None of the above or other (4)  

Provider training + education

Q5 Have you received...

**Table 2:** Q5 Have you received...

	Yes (1)	No (2)
Formal family planning training in residency/before graduating from training? (1)	o	o
Contraceptive training in residency/before graduating from training (i.e. IUD insertion training) (2)	o	o
Any formal education/training about over-the-counter birth control pills? (3)	o	o
Information from your institution/practice about over-the-counter birth control pills? (4)	o	o

Q6 Where do you receive new information about birth control? Please select all that apply.
 

▢        Medical literature (i.e. The Green Journal) (1)  

▢        Medical-targeted websites (i.e. WebMD) (2)  

▢        Social media (i.e. Facebook, Instagram) (3)  

▢        Continuing Medical Educatoin (CME) (4)  

▢        In-house education or training (5)  (5)

▢        Supplementary resources (i.e. Bedsiders) (6)  (6)

Appendix B: Perceptions of patient volume/demographics

Q7 Approximately what portion of your patient population...
 

**Table 3:** Q7 Approximately what portion of your patient population...

	None (1)	Some (2)	Most (3)	Nearly all (4)
Receives birth control counseling from you/your institution? (1)	o	o	o	o
Experiences unintended pregnancies? (2)	o	o	o	o
Have no medical insurance? (3)	o	o	o	o
Are Medicaid recipients? (4)	o	o	o	o
Identifies as Black? (5)	o	o	o	o
Identifies as Hispanic or Latinx? (6)	o	o	o	o
Is underserved or has limited access to health care? (7)	o	o	o	o

Appendix C: Provider perception of birth control barriers

Q8 How many of your patients have reported...

**Table 4:** Q8 How many of your patients have reported...

	None (1)	Some (2)	Most (3)	Nearly all (4)
Difficulty accessing birth control (i.e. filling prescriptions, obtaining appointments) (1)	o	o	o	o
A lack of understanding of how to use their birth control (i.e. timing of pills, replacing IUDs) (2)	o	o	o	o
Becoming pregnant despite using birth control? (3)	o	o	o	o
Interest or curiosity regarding the Opill? (4)	o	o	o	o

Appendix D: Comfortability

Q9 How comfortable are you...

**Table 5:** Q9 How comfortable are you...

	Very uncomfortable (1)	Somewhat uncomfortable (2)	Somewhat comfortable (3)	Very comfortable (4)
Counseling on birth control? (1)	o	o	o	o
Prescribing progestin-only pills for birth control (for patients without contraindications)? (2)	o	o	o	o

Appendix E: Prescribing Opill

Q10 Are you aware that the Opill is currently available over-the-counter?

o Yes (1)  

o No (2)  

Q11 If so, through what source? Please select all that apply.

▢        Medical literature (i.e. The Green Journal) (1)  

▢        Medical-targeted websites (i.e. WebMD) (2)  

▢        Social media (i.e. Facebook, Instagram) (3)  

▢        Continuing Medical Education (CME) (4)  

▢        In-house education or training (5)  

▢        Supplementary resources (i.e. Bedsiders) (6)  

▢        Other (7)  __________________________________________________

Q12 How often do you prescribe or counsel patients about the Opill?

o Very often (multiple times per week) (1)  

o Often (about once per week) (2)  

o Sometimes (once per month) (3)  

o Rarely (few times per year) (4)  

o Never (5)  

Q13 How often do you discourage patients from using the Opill?

o Very often (multiple times per week) (1)  

o Often (about once per week) (2)  

o Sometimes (once per month) (3)  

o Rarely (few times per year (4)  

o Never (5)  

Q14 Do you recommend Opill to your patients? Why or why not?

o I recommend Opill to my patients. (1) 

 __________________________________________________

o I do not recommend Opill to my patients. (2)   __________________________________________________

o I am unsure about recommending Opill to my patients. (3)   __________________________________________________

Q15 Which patients would you recommend Opill to? Select all that apply.

▢        All my patients that seek a contraceptive to prevent unintended pregnancies, given they do not have a contraindication to Opill. (1)  

▢        Patients that experience barriers in seeing their provider (financial, transportation, language, etc.) (2)  

▢        Patients that experience unwanted side effects from the combined pill or other birth control methods. (3)  

▢        None of my patients. (4)  

Q16 Which patients do you foresee having issues with the Opill as a birth control method? Select all that apply.

▢  All patients seeking an oral contraceptive. (1)  

▢  Patients who lack adherence to daily ingestion of oral contraceptives.   

▢  Patients with financial barriers to pay for Opill over-the-counter. (3)  

▢  Patients with contraindications to Opill such as breast cancer. (4)  

▢  No patients seeking a contraceptive to prevent unintended pregnancies. (5) 

Q17 How often do you prescribe other progestin only pills?

o Very often (multiple times per week) (1)  

o Often (about once per week) (2)  

o Sometimes (once per month) (3)  

o Rarely (few times per year) (4)  

o Never (5)  

Q18 Do you suggest only prescription oral contraceptives for patients? Why or why not?

o Yes (1)   __________________________________________________

o No (2)   __________________________________________________

o Unsure (3)  __________________________________________________

Appendix F: Concerns with Opill

Q19 Please indicate your agreement with the following statements:

**Table 6:** Q19 Please indicate your agreement with the following statements:

	Strongly disagree (1)	Somewhat disagree (2)	Somewhat agree (3)	Strongly agree (4)
I would recommend an over-the-counter progestin-only pill for patients without contraindications seeking birth control. (1) (	o	o	o	o
Many of my patients would have a medical contraindication to an over-the-counter progestin-only pill. (2)	o	o	o	o
Many of my patients would have difficulty accessing an over-the-counter progestin-only pill. (3)	o	o	o	o

Q20 Do you have concerns with an over-the-counter progestin only birth control pill?

o Yes  (1)

o No  (2)

o Unsure at this time  (3)

Q21 What are your concerns regarding the use of Opill in your patient population? If none, write "none". 

________________________________________________________________

Appendix G: POP misconceptions

Q22 Please indicate your agreement with the following statements:
 

**Table 7:** Q22 Please indicate your agreement with the following statements:

	False (1)	True (2)
Progestin-only birth control is equally effective at preventing unwanted pregnancy than combination birth control when both are taken correctly. (1)	o	o
Extensive literature has shown that progestin-only birth control is associated with a much higher incidence of weight gain than combination pills. (2)	o	o
Adherence to progestin-only pills due to their limited efficacy outside of a 3-hour window is significantly lower than adherence to daily oral contraceptives. (3)	o	o
Progestin-only pills have a higher occurrence of contraindications compared to oral contraceptives. (4)	o	o
Patients are uninterested in switching from prescribed oral contraceptives to OTC progestin-only pills. (5)	o	o
Part of Opill's mechanism for preventing untintended pregancies is by thickening the cervical mucus. (6)	o	o
